# Tribbles 3 Regulates the Fibrosis Cytokine TGF-***β***1 through ERK1/2-MAPK Signaling Pathway in Diabetic Nephropathy

**DOI:** 10.1155/2014/240396

**Published:** 2014-07-16

**Authors:** Luwei Zhang, Jinhang Zhang, Xinnong Liu, Shengli Liu, Jun Tian

**Affiliations:** ^1^Department of Hemodialysis, Qilu Hospital, Shandong University, Jinan 250012, China; ^2^General Department, Qilu Hospital, Shandong University, Jinan 250012, China

## Abstract

To reveal the expression and possible role of tribbles homolog 3 (TRB3) in the incidence of type 2 diabetic nephropathy, we used immunohistochemistry, real-time quantitative PCR, western blot analysis, and enzyme-linked immunosorbent assay (ELISA) to study the expression of TRB3, extracellular signal-regulated kinase 1/2 mitogen-activated protein kinase (ERK1/2 MAPK), transforming growth factor *β*1 (TGF-*β*1), and collagen type IV in kidneys of db/db diabetic mice and in murine renal mesangial cells stimulated with high glucose. The expression of TRB3, TGF-*β*1, and collagen type IV was increased in kidneys of db/db diabetic mice. TGF-*β*1 and collagen type IV regulated by high glucose through ERK1/2 MAPK were downregulated by silencing TRB3 in renal mesangial cells. TRB3 may be involved in diabetic nephropathy by regulating the fibrosis cytokine TGF-*β*1 and collagen type IV through the ERK1/2 MAPK signaling pathway.

## 1. Introduction

Diabetic nephropathy (DN) is an important diabetic microvascular complication and the major cause of disability and death. Morbidity and mortality with the disease are increasing every year [1]. DN is characterized by albuminuria, glomerular hypertrophy, and progressive accumulation of glomerular matrix, culminating in glomerulosclerosis, tubulointerstitial fibrosis, and progressive loss of renal function [2, 3].

Many studies have confirmed that glomerular sclerosis and interstitial fibrosis are the main pathologic characteristics in DN, especially in the midanaphase of DN. Deposition of extracellular matrix (ECM) such as collagens and fibronectin [4] regulated by transforming growth factor *β*1 (TGF-*β*1) is the core mechanism of glomerular sclerosis and interstitial fibrosis. The mesangial cells play important roles in DN, being responsible for the accumulation of ECM and mesangial expansion [5]. TGF-*β*1 is the core cytokine leading to the synthesis of ECM, which is responsible for mesangial fibrosis and hypertrophy under diabetic conditions [6]. The major components of the ECM proteins collagen types I–IV and their synthesis and immoderate deposition are consistently observed in multifarious renal disease processes affecting humans and experimental animals [7, 8].

Tribbles homolog 3 (TRB3) is an important member of the tribbles family. Combined with unphosphorylated Akt, TRB3 can prevent Akt activity and negatively regulate the insulin signaling pathway [9]. TRB3 and its gene polymorphism are associated with insulin resistance, a vital pathophysiologic characteristic of type 2 diabetes. TRB3 also serves as a scaffold protein and regulates the activation of the three classes of mitogen-activated protein kinases (MAPKs) [10]. As a member of the MAPK family, extracellular signal-regulated kinase 1/2 (ERK1/2) can be activated in mesangial cells exposed to high glucose (HG) [11]. Also, ERK activity may enhance the TGF-*β*1-dependent responses in human mesangial cells [12]. Therefore, TRB3 may be involved in DN through an ERK pathway.

In this study, we aimed to explore the role of TRB3 in DN and the possible regulating mechanism between TRB3, ERK, and TGF-*β*1* in vivo* and* in vitro*.

## 2. Materials and Methods

### 2.1. Main Reagents

We obtained antibodies for TRB3 (Santa Cruz Biotechnology, Santa Cruz, CA), TGF-*β*1 (Abcam Biotechnology, CA), and PD98059, phospho-ERK1/2, and total ERK1/2 (Cell Signaling Technology, Beverly, MA). Trizol reagent and reagents for RT-PCR were from Takara Biotechnology (Dalian, China). ELISA kits for collagen types I and IV were from R&D systems (Minneapolis, MN).

### 2.2. Experimental Animals

SPF db/db diabetic mice (C57BL/KSJ) and their matched (12-week-old) controls (db/m) were obtained from Vital River Laboratory Animal Technology (Beijing). All animals were maintained on a normal diet under standard animal house conditions at the cardiovascular remodeling Laboratory Animal Center in Qilu Hospital of Shandong University. Animal experiments were conducted in accordance with guidelines established by the Animal Care and Use Committee of Shandong University.

Animals were divided into 3 groups (*n* = 5) and killed at 16, 20, and 25 weeks. Blood glucose and body weight were randomly monitored weekly; levels of urinary albumin excretion, serum creatinine, and blood urea nitrogen (BUN) were detected before death once. The left kidney pieces fixed in 4% paraformaldehyde were embedded in paraffin, sectioned at 4 *μ*m thickness, and mounted on glass slides. The slides were used for morphological observation and immunohistochemical staining to detect the expression of TRB3; the right kidney was snap-frozen in liquid nitrogen and stored at −80°C for RT-PCR and western blot analysis.

### 2.3. Histology

Sections were stained with hematoxylin and eosin (H&E), periodic acid Schiff (PAS), and Masson trichrome for investigating renal pathological changes. Paraffin sections were dewaxed and washed in phosphate buffered saline (PBS) and then incubated in preheated 10 mmol/L sodium citrate buffer at 94°C for 15 min. Slides were washed and blocked with protein-blocking solution (10% normal goat serum) for 30 min and then incubated with rabbit anti-mouse TRB3 antibody (1 : 100) overnight at 4°C, biotin-labeled secondary antibody working solution for 30 min at 37°C, and then DAB color. Sections were preincubated with PBS as negative controls.

### 2.4. Cell Culture

Mycoplasma-free SV40 MES 13 cells (murine mesangial cells, MMCs) were purchased from China Center for Type Culture Collection. They were derived from glomerular explants of SV40 transgenic mice and showed both biochemical and morphological features of normal mesangial cells in culture. They were maintained in DMEM-F12 (3 : 1) containing 6 mM glucose and supplemented with 14 mM HEPES and 5% fetal bovine serum in 5% CO_2_ and 95% humidified air at 37°C as described previously [13, 14]. Cells were synchronized by culturing in serum-free medium for 24 h before testing and all experiments were performed with cells between passages 30 and 40 to minimize the effects of phenotypic variation in continuous culture.

Mesangial cells were divided by glucose concentration into 3 groups: (1) normal glucose (NG, 5.6 mM glucose, and control); (2) HG (25 mM glucose); and (3) NG + high mannitol (HM, NG plus 19.5 mM mannitol). To investigate the effects of glucose on the expression of TRB3 and pERK1/2, cells were stimulated with HM or HG for 6, 12, 24, and 48 h. At the end of each time, total RNA and protein of the cells were extracted for TRB3 and pERK1/2 expression.

To examine the effect of the MAPK pathway on collagen expression by HG, a specific ERK inhibitor (PD98059, 10 *μ*mol/L) was added 1 h before stimulation. To examine the effect of TRB3 on collagen expression by HG, TRB3 small interfering RNA (siRNA) was transfected 6 h before stimulation and cultured for 48 h in NG or HG medium; then, total RNA and protein were extracted from cells for analysis of TRB3, TGF-*β*1, and pERK1/2 expression and culture medium was collected for measurement of concentration of collagen types I and IV.

### 2.5. siRNA Transfection

Cells were replated and transfected in 6-well plates with 150 *μ*L Opti-MEM (Invitrogen, CA) and 1.5 *μ*L/well Lipofectamine 2000 (Invitrogen, CA) with 20 pmol siRNA and its controls. The sense and antisense sequences of the primers were 5′-GCACAGAGUACACCUGCAATT-3′ and 5′-UUGCAGGUGUACUCUGUGCTT-3′. As a negative control, we used randomly mixed sequences of TRB3 siRNA, 5′-UUCUCCGAACGUGUCACGUTT-3′, and 5′-ACGUGACACGUUCGGAGAATT-3′. The effect of siRNA knockdown of TRB3 on the expression of TGF-*β*1 and collagen types I and IV was evaluated by western blot analysis or ELISA at 48 h. All RNAi experiments were repeated at least 3 times.

### 2.6. Real-Time Quantitative PCR

Total RNA was extracted by use of Trizol. In total, 1 *μ*g total RNA was reverse-transcribed in a 20 mL volume containing 0.5 mg oligo-dT primer, 1 mL dNTP mixture, 1.25 mL RNase inhibitor, and 4 U reverse transcriptase. Real-time quantitative PCR involved the SYBR-based method in a 20 mL reaction in a Roche light-cycler. Reaction specificity was confirmed by analyzing melting curves and by electrophoresis on 2.0% agarose gel analysis of products. The relative change in gene expression was analyzed by the 2^−ΔCt^ method and normalized to the expression of the housekeeping gene *β*-actin. Respective primer and product specifications are in Table 1.

### 2.7. Western Blot Analysis

Total protein was extracted from tissues and cells as described previously. The protein extracts were separated on 10% SDS-PAGE then transferred to PVDF membranes, and blocked in TBST with 5% skim milk at room temperature for 2 h. The blots were incubated with antibodies for TRB3 (1 : 200), TGF-*β*1 (1 : 200), pERK1/2 (1 : 2000), ERK1/2 (1 : 1000), and *β*-actin (1 : 1000) overnight at 4°C, washed, and then incubated with goat anti-rabbit antibody (1 : 10000) at room temperature for 1 h. Protein bands were analyzed by use of AlphaView SA software. All experiments were repeated at least 3 times.

### 2.8. ELISA

Soluble collagen types I and IV proteins were determined by ELISA kits according to the manufacturer's protocol. The kits for mouse collagen types I and IV were species-specific and sensitive up to 1000, 10, and 20 ng/mL. All concentrations of proteins were normalized to the total protein amount.

### 2.9. Statistical Analyses

All values were expressed as means ± SEM. Images were analyzed by use of Image-Pro Plus 6.0 for semiquantitative analysis. Groups were compared by one-way ANOVA and correlation analysis involved Pearson correlation coefficient. Statistical analysis involved SPSS v17.0 for Windows (SPSS Inc., Chicago, IL). *P* < 0.05 was considered statistically significant.

## 3. Results

### 3.1. Evaluation of the DN Mouse Model

**Figure 1:**
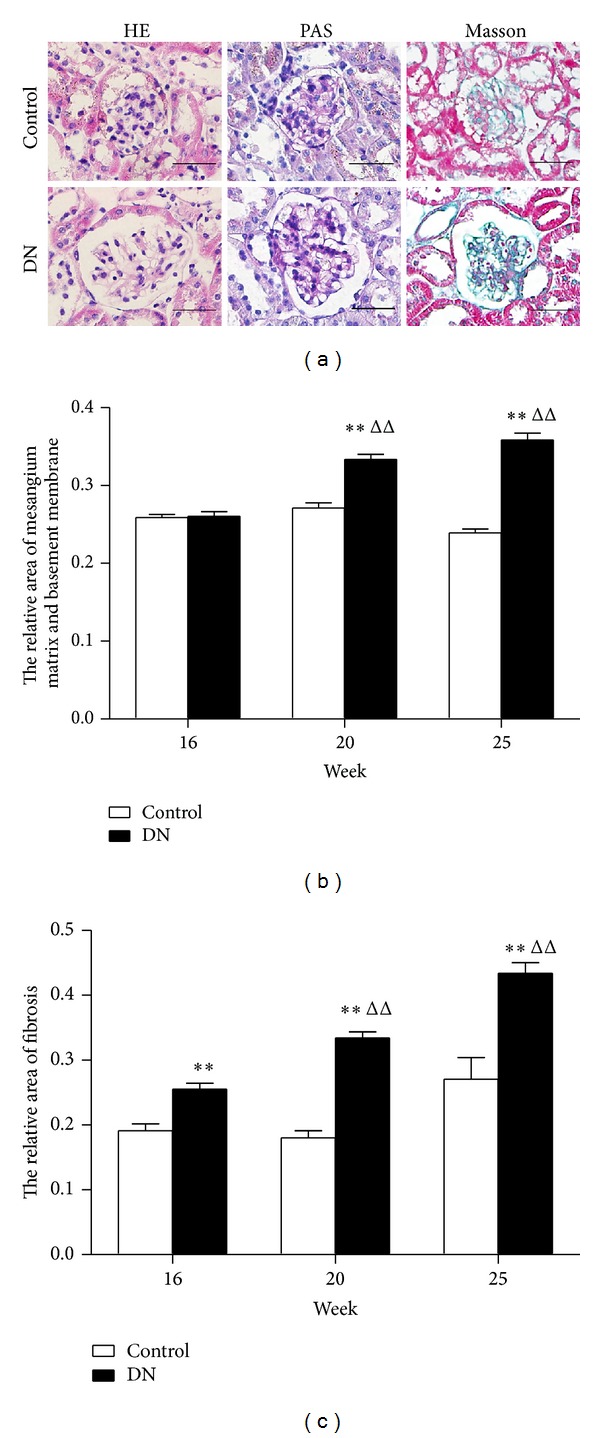
Glomerular pathological changes in mice with diabetic nephropathy (DN) and control mice. (a) Renal morphology and glycogen accumulation at 25 weeks evaluated by hematoxylin and eosin (HE) and periodic acid Schiff (PAS) staining, renal interstitial fibrosis detected by Masson trichrome staining. (b) Relative area of mesangium matrix and basement membrane at different times. (c) Relative area of renal interstitial fibrosis at different times. In both experiments, more than 12 glomeruli were evaluated in each mouse. Magnification in (a) HE, PAS, Masson: 400x. *P < 0.05, **P < 0.01 versus age-matched control mice. ^Δ^ *P* < 0.05, ^ΔΔ^ *P* < 0.01 versus 16-week-old DN mice. Scale bars, 50 *μ*m. *n* = 5 mice per group.

The blood glucose results for control mice remained stable, whereas DN mice showed hyperglycemia. Levels of UAE, Scr, and BUN and body weight were higher for db/db mice than age-matched controls (*P* < 0.05, *P* < 0.01) (Table 2). Mesangial matrix expansion and mesangial area were wider for DN than control mice. At 25 weeks, DN mice showed diffuse and nodular mesangial sclerosis (Figure 1(a)), and the area of mesangium matrix and basement membrane was increased (Figure 1(b)) as was the relative fibrosis area (Figure 1(c)).

### 3.2. TRB3 Expression Increased in Kidney of DN Mice

**Figure 2:**
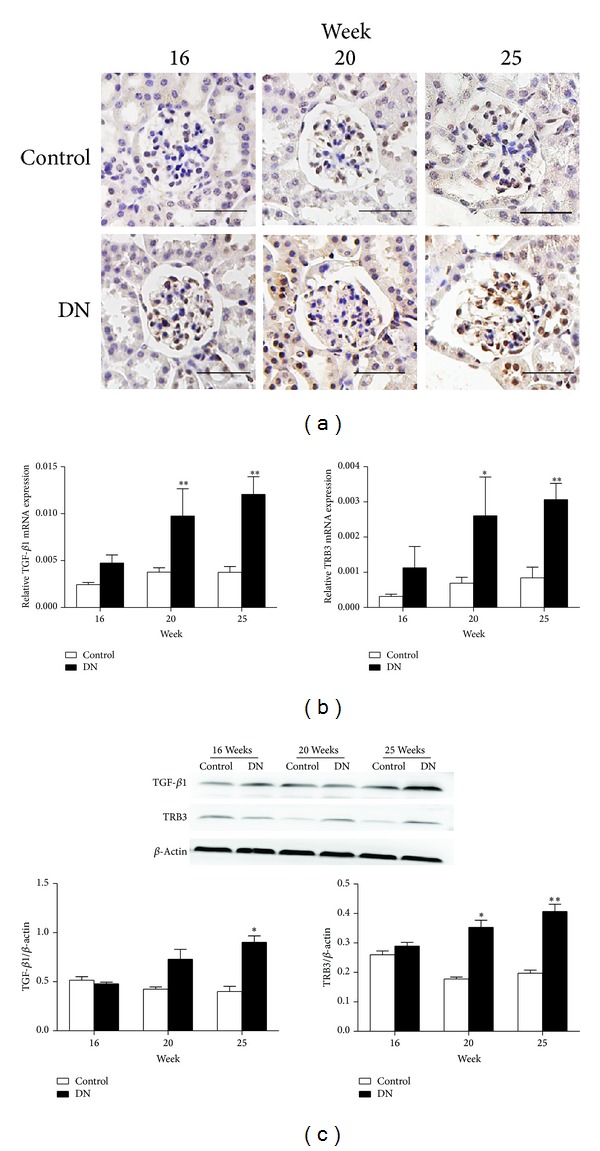
mRNA and protein expression of TGF-*β*1 and TRB3 upregulated in DN mice. (a) Protein TRB3 level in nucleus of intrinsic glomerular cells and tubular epithelial cells. Total TGF-*β*1 and TRB3 mRNA (b) and protein (c) levels by RT-PCR and western blot analysis, respectively. *P < 0.05, **P < 0.01 versus age-matched control mice. Scale bars, 50 *μ*m. *n* = 5 mice per group.

TRB3 was expressed mainly in the nucleus of intrinsic glomerular cells and tubular epithelial cells (Figure 2(a)). The expression of TRB3 was higher in DN than control mice. The mRNA and protein expression of TRB3 and TGF-*β*1 were higher in DN than control mice from 20 weeks and increased with time (Figures 2(b) and 2(c)). The protein level of TRB3 was positively correlated with TGF-*β*1 level (*r* = 0.944, *P* < 0.01) and renal interstitial fibrosis (*r* = 0.857, *P* < 0.05 in DN mice).

### 3.3. Effect of HG on the Expression of TRB3 in MMCs

**Figure 3:**
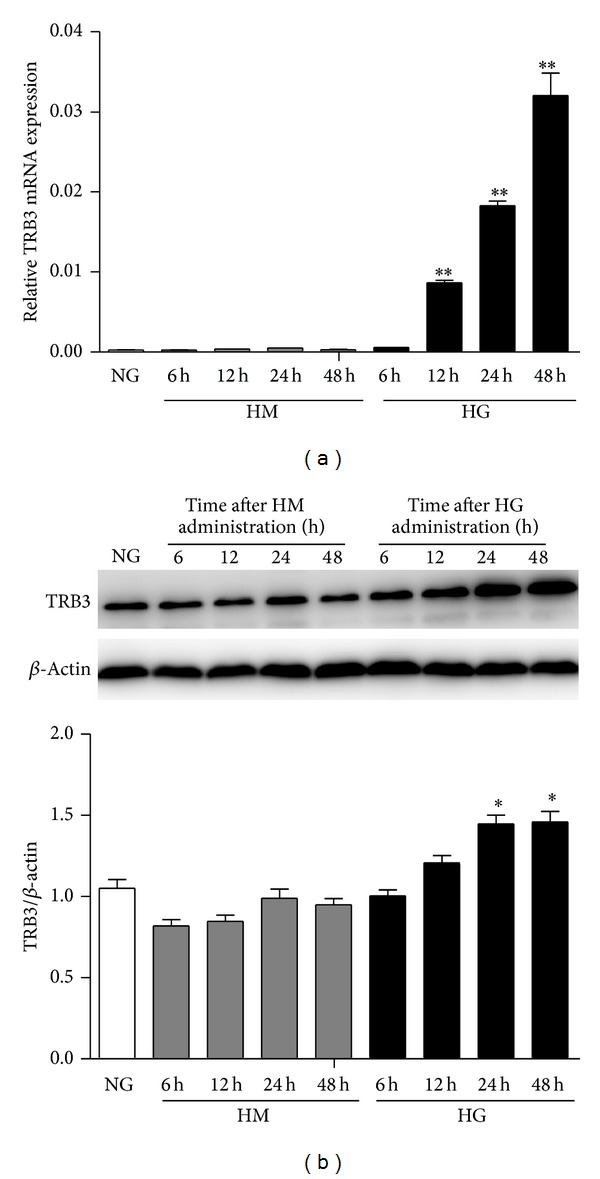
Effect of high glucose (HG) on TRB3 mRNA and protein levels in murine mesangial cells (MMCs) over time. MMCs were cultured in media containing NG and then stimulated with NG + high mannitol (HM) or HG for 6, 12, 24, and 48 h. (a) RT-PCR analysis of the mRNA level of TRB3. (b) Western blot analysis of the protein level of TRB3. Data are mean ± SEM. *P < 0.05, **P < 0.01 versus NG.

To confirm the effect of glucose on the expression of TRB3, MMCs were stimulated with HG or HM for various times. The mRNA level of TRB3 was increased within 12 h after HG stimulation and peaked at 48 h (*P* < 0.01, versus NG; Figure 3(a)). TRB3 protein level was increased under HG at 12, 24, and 48 h (Figure 3(b)). This increase also peaked at 48 h (*P* < 0.05, versus NG). However, levels did not increase significantly under HM at different times. Thus, HG can upregulate the expression of TRB3 in MMCs.

### 3.4. HG Upregulated the Expression of TGF-*β*1 and Collagen Type IV in Cultured Cells

**Figure 4:**
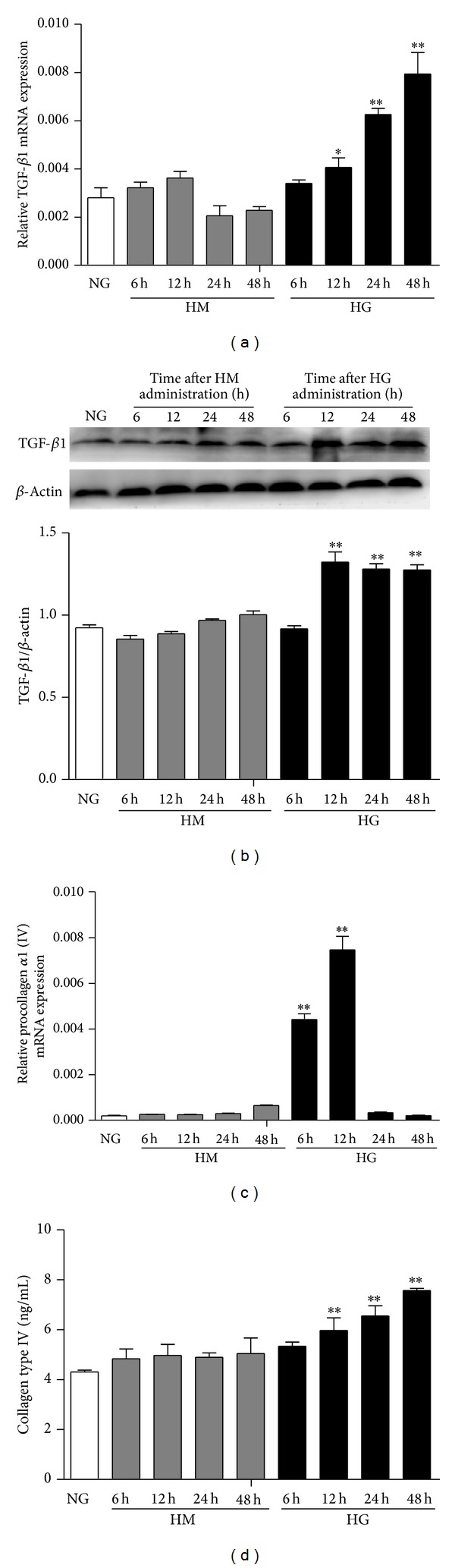
Effect of HG on mRNA and protein levels of TGF-*β*1 and collagen type IV in MMCs over time. MMCs were cultured in media containing NG and then stimulated with HM or HG for 6, 12, 24, and 48 h. (a) and (c) RT-PCR analysis of mRNA levels of TGF-*β*1 and collagen type IV, respectively. (b) Western blot analysis of protein level of TGF-*β*1. (d) ELISA of secretion of collagen type IV. Data are mean ± SEM. *P < 0.05, **P < 0.01 versus NG.

As compared with NG, HG time-dependently increased both the mRNA and protein expression of TGF-*β*1 and collagen type IV (Figure 4) within 48 h and 12 h, respectively, for mRNA and for both within 48 h for protein (*P* < 0.01, versus NG). However, collagen type I expression did not change under any conditions within 48 h (data not shown). Therefore, HG increased TGF-*β*1 and collagen type IV secreted from cultured MMC cells.

### 3.5. Effect of TRB3 on Activation of the ERK1/2 MAPK Pathway in MMCs

**Figure 5:**
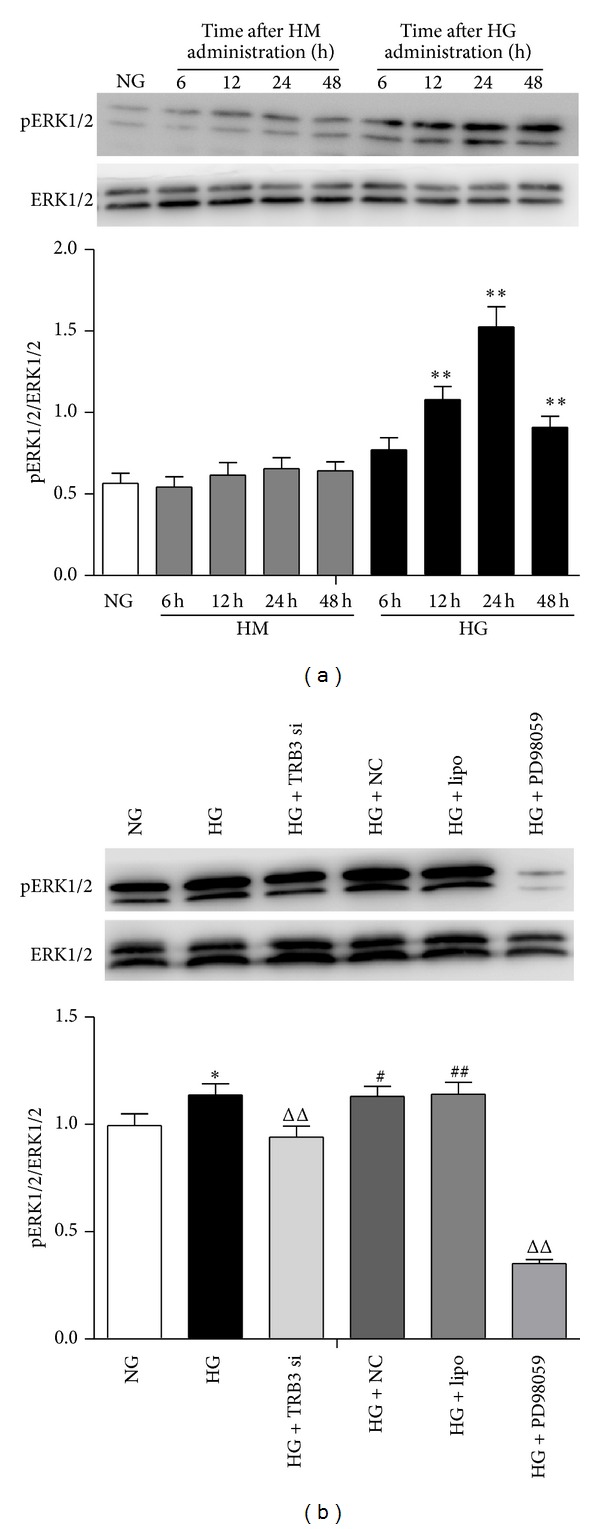
Effect of TRB3 on phosphorylated ERK1/2 (pERK1/2) expression in cells. (a) Western blot analysis of pERK1/2 level in cells incubated with HM or HG for 6, 12, 24, and 48 h. (b) Cells were transfected with TRB3 siRNA or corresponding negative control. Western blot analysis of pERK1/2 level with HG stimulation for 48 h. Data are mean ± SEM. *P < 0.05, **P < 0.01 versus NG, ^ΔΔ^ *P* < 0.01 versus HG, and ^#^ *P* < 0.05, ^##^ *P* < 0.01 versus NG +TRB3 siRNA.

**Figure 6:**
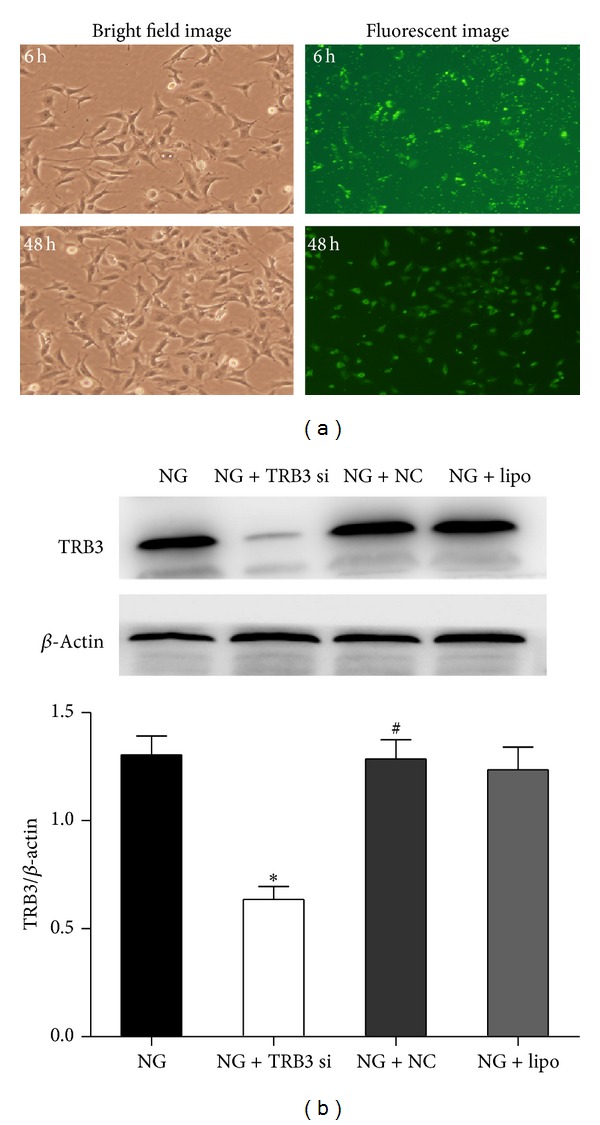
Effect of siRNA on the expression of FAM and TRB3 in cells. Transient transfection of cells with TRB3 siRNA induced FAM expression, indicating successful transfection. FAM expression was observed at 0 and 48 h after transfection. Cells were transfected with TRB3 siRNA or its corresponding negative control and then incubated with NG (5.5 mM glucose) for 48 h. (a) FAM expression was observed by bright field and fluorescent imaging (200x). (b) Western blot analysis of the protein expression of TRB3 at 48 h after TRB3 siRNA transfection. Data are mean ± SEM. *P < 0.05 versus NG; ^#^ *P* < 0.05 versus NG +TRB3 siRNA.

To verify the effect of glucose on the activation of the ERK1/2 MAPK pathway in MMCs, cells were cultured in NG medium and then stimulated with HG or HM for various times. The level of pERK1/2 increased during the first 6 h (*P* < 0.01, versus NG) after HG stimulation and peaked at 24 h (*P* < 0.01, versus NG) (Figure 5(a)). However, stimulation with HM had no effect on the activation of this pathway. Therefore, HG can activate the ERK1/2 pathway in MMCs. To confirm the effect of TRB3 on this pathway, we transfected TRB3 siRNA into MMCs exposed to HG medium for 24 h and evaluated pERK1/2 levels. Transient transfection of siRNA into MMCs induced FAM expression, which indicated successful transfection. Expression of FAM increased at 6 h and peaked at 48 h (Figure 6(a)). To test the efficacy of the selected siRNA sequence, we measured the protein level of TRB3 after 48 h transfection with TRB3 siRNA. TRB3 protein expression was lower in MMCs with TRB3 siRNA than in cells with control siRNA (*P* < 0.05, versus NG + NC) (Figure 6(b)). pERK1/2 expression was decreased in MMCs transfected with TRB3 siRNA (*P* < 0.05, versus HG + NC; Figure 5(b)). TRB3 may activate the ERK1/2 MAPK pathway in MMCs.

### 3.6. TRB3 is Involved in the Expression of TGF-*β*1 and Collagen Type IV Regulated by High Glucose in DN by Activating ERK1/2 MAPK

**Figure 7:**
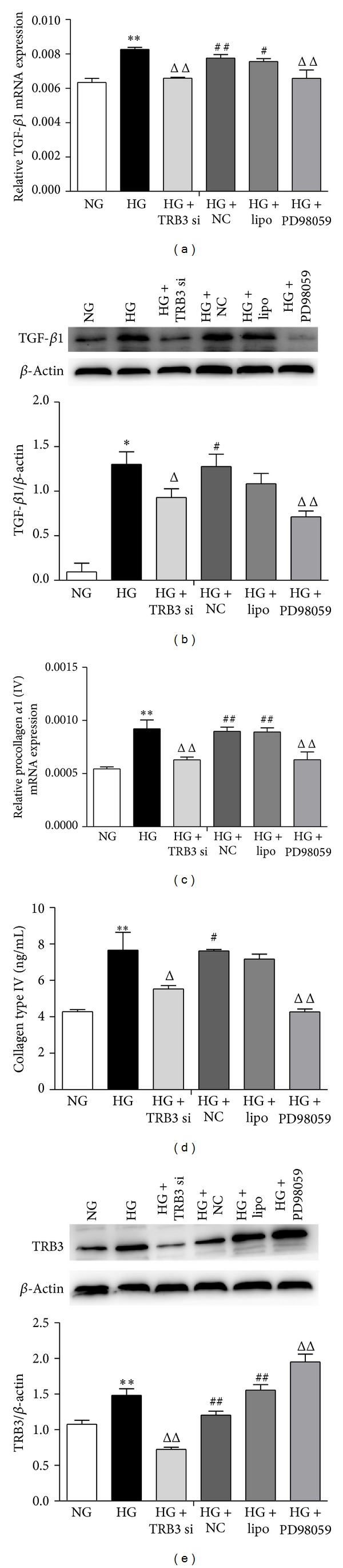
Effect of silencing TRB3 protein or blocking ERK1/2 MAPK pathway by a specific ERK inhibitor (PD98059, 10 *μ*mol/L) on HG induction of TGF-*β*1, collagen type IV, and TRB3 level in MMCs. (a) and (b) mRNA level of TGF-*β*1 at 12 h and protein level at 48 h; (c) and (d) mRNA level of collagen type IV at 12 h and protein level at 48 h. MMCs were stimulated with NG or HG after blockade of TRB3 with TRB3 siRNA. (e) Protein expression of TRB3 at 48 h. Data are mean ± SEM. *P < 0.05, **P < 0.01 versus NG; ^Δ^ *P* < 0.05, ^ΔΔ^ *P* < 0.01 versus HG; ^#^ *P* < 0.05, ^##^ *P* < 0.01 versus NG + TRB3 siRNA.

The expression of pERK1/2 was markedly reduced by blocking ERK1/2 MAPK signaling by a specific ERK inhibitor (PD98059, 10 *μ*mol/L) (Figure 5(b)). The mRNA and protein levels of TGF-*β*1 and collagen type IV were decreased with the ERK1/2 pathway blocked (Figures 7(a)–7(d), *P* < 0.01 versus HG) as was the pERK1/2 level (Figure 5(b), *P* < 0.01 versus HG), which suggests that HG can regulate TGF-*β*1 and collagen type IV through the ERK1/2 MAPK pathway. The protein expression of TRB3 was markedly increased after blocking the ERK1/2 MAPK pathway (Figure 7(e), *P* < 0.01 versus HG). This finding indicates a possible feedback regulation between TRB3 and some downstream cytokines of ERK1/2 MAPK.

## 4. Discussion

Here, we found augmented expression of TRB3 in kidneys of DN mice, which was positively related to TGF-*β*1 expression and renal interstitial fibrosis. HG upregulated the expression of TRB3, TGF-*β*1, collagen type IV, and phosphorylated-ERK1/2 MAPK in MMCs. After inhibiting TRB3 with TRB3 siRNA, the HG-induced level of pERK1/2 MAPK was attenuated and HG-induced expression of TGF-*β*1 and collagen type IV decreased. Moreover, the protein expression of TRB3 was increased after blocking the ERK1/2 MAPK pathway. TRB3 may be involved in DN by regulating the fibrosis cytokine TGF-*β*1 and collagen type IV via ERK1/2 MAPK signaling.

Tribbles was first discovered to regulate Drosophila embryogenesis, and it has the same location as the type 2 diabetes gene, so TRB3 may have a natural link with diabetes. Compared with wild-type mice, db/db diabetic mice were found to have increased TRB3 mRNA and protein expression in liver; TRB3 expression increased in the db/db mouse liver promoted blood glucose and increased glucose tolerance [9]. However, few studies have reported the expression and role of TRBs in DN. We examined the expression of TRB1, TRB2, and TRB3 in the DN mouse kidney and found only TRB3 expressed differently between DN and control groups, so TRB3 could be a potential cytokine to widen the current knowledge of DN. Our further study revealed that the mRNA and protein levels of TRB3 were higher in the DN than normal kidney, which was also positively correlated with TGF-*β*1 protein level and content of collagen. Previous study showed that TRB3 was positively correlated with kidney tissue fibrosis, which may play a role in promoting the progression of fibrosis by inducing the transformation between epithelial and mesenchymal tissue [15]. Therefore, TRB3 may be involved in DN by inducing interstitial fibrosis, in which TGF-*β*1 plays a key role.

We wondered about the role of TRB3 in the fibrosis of DN and the relationship between TRB3 and TGF-*β*1. Several studies supported that TRB3 may function as a scaffold protein to control MAPK activity [10, 16, 17]. Among the several MAPK signal pathways, the ERK1/2 pathway is activated under HG in mesangial cells, followed by the complicated synthesis of TGF-*β*1 [18]. The activation of the ERK1/2 pathway is necessary for HG-induced production of TGF-*β*1 and connective tissue growth factor (CTGF) in MMCs [19]. Our preliminary study excluded the involvement of the p-38 and JNK MAPK pathway. Therefore, we focused on the ERK MAPK pathway. Silencing TRB3 decreased ERK1/2 activation, followed by decreased mRNA and protein levels of TGF-*β*1 and collagen type IV in MMCs, so TRB3 may participate in renal fibrosis of DN by upregulating TGF-*β*1 and collagen type IV in MMCs via ERK MAPK signaling. To our knowledge, our study is the first to reveal the interaction between TRB3, ERK1/2, and TGF-*β*1 in renal tissue of DN.

Recently, TRB3 was found stimulated in diabetic kidneys, regulated by the ER stress marker CHOP, and inhibited the podocyte expression of monocyte chemoattractant protein 1, which first suggested that TRB3 plays a protective role in diabetic kidney disease [20]. These findings that differ from our results may be due to cell specificity [21]; we used mesangial cells, but the previous study used podocytes.

Moreover, signal transduction* in vivo *is a complex system. Numerous cytokines may interact through different pathways, and different signal pathways may have “crosstalk.” So, the role of TRB3 in DN can also be studied from other signal pathways. TRB3 plays a role in the pathogenesis of DN by participating in insulin resistance, functioning as a negative modulator of Akt [9, 22, 23]. Another study showed that TRB3 may cause renal cell apoptosis by participating in the NF-*κ*B pathway and thus participate in the process of renal fibrosis in DN [24, 25]. Even Smad3 is considered to mediate TRB3 enhancing TGF-*β* signaling [26]. There must be some interactions between ERK, Akt, NF-*κ*B, Smad3, and other unknown pathways. More detailed and systemic studies are needed to establish the complete theory on TRB3 involved in DN.

In this study, we found that the expression of TRB3 increased after blocking the ERK1/2 MAPK pathway, which may indicate a negative feedback regulation between TRB3 and some downstream cytokines of ERK1/2 MAPK or special “crosstalk” with other signal pathways, which requires further exploration.

## 5. Conclusions

TRB3 expression is upregulated in renal tissue of DN mice* in vivo* and MMCs* in vitro*. TRB3 may be involved in DN by regulating the fibrosis cytokine TGF-*β*1 and collagen type IV via ERK1/2 MAPK signaling.

## Figures and Tables

**Table 1:** Primers used for RT-PCR.

Name	GenBank accession	Primers	Size (bp)
TRB3	NM 175093.2	Forward: 5′-GCTCTGAGGCTCCAGGACAA-3′	91
		Reverse: 5′-TGTCATCAAACTCCAACGGTTTC-3′	

TGF-*β*1	NM 011577.1	Forward: 5′-GTGTGGAGCAACATGTGGAACTCTA-3′	174
		Reverse: 5′-CGCTGAATCGAAAGCCCTGTA-3′	

Collagen type I *α*1	NM 007742.3	Forward: 5′-CAACAGTCGCTTCACCTACAGC-3′	201
		Reverse: 5′-GTGGAGGGAGTTTACACGAAGC-5′	

Collagen Type IV *α*1	NM 009931.2	Forward: 5′-TATGTCCAAGGCAACGAGC-5′	228
		Reverse: 5′-AACCGCACACCTGCTAATG-3′	

*β*-Actin	NM 007393.3	Forward: 5′-GTG ACG TTG ACA TCC GTA AAG A-3′	245
		Reverse: 5′-GCC GGA CTC ATC GTA CTC C-3′	

**Table 2:** Metabolic data of mice in different groups by time.

	16 weeks		20 weeks		25 weeks	
	C	DN	C	DN	C	DN
BW (mg)	22.80 ± 2.52	47.86 ± 0.83**	22.54 ± 1.24	52.26 ± 2.47**	23.26 ± 0.67	47.52 ± 2.56**
RBG (mmol/L)	4.48 ± 0.71	12.33 ± 1.67**	4.78 ± 0.84	24.15 ± 1.62^∗∗△^	5.92 ± 0.87	33.08 ± 1.54^∗∗△^
UAE (*μ*g/24 h)	27.68 ± 1.65	386.12 ± 16.30**	24.20 ± 1.45	452.12 ± 32.26^∗∗△^	27.24 ± 1.52	551.78 ± 29.19^∗∗△△^
BUN (mmol/L)	8.32 ± 0.37	9.38 ± 0.29*	8.56 ± 0.43	9.94 ± 0.20**	8.44 ± 0.45	10.48 ± 0.18^∗∗△^
Scr (mmol/L)	14.80 ± 0.58	28.2 ± 1.7	18.00 ± 1.30	33.60 ± 3.37**	16.60 ± 1.67	25.40 ± 2.77**

Data are means ± SEM. C: control; DN: diabetic nephropathy; BW: body weight; UAE: urinary albumin excretion; BUN: blood urea nitrogen; Scr: serum creatinine.

*P < 0.05, **P < 0.01 versus age-matched control mice.

^△^ *P* < 0.05, ^△△^ *P* < 0.01 versus 16-week-old db/db mice.
